# Acute Effects of Inspiratory Muscle Warm-Up on Performance and Cardiorespiratory Parameters of Scuba Divers—A Preliminary Study

**DOI:** 10.3390/jfmk10020105

**Published:** 2025-03-26

**Authors:** Ricardo Alberola-Blanes, Fernando Alacid, Carmen Daniela Quero-Calero, Daniel López-Plaza

**Affiliations:** 1 Department of Education, Health Research Center, University of Almeria, 04120 Almeria, Spain; rickyalberola@gmail.com; 2 International Chair of Sport Medicine, UCAM Catholic University of Murcia, 30007 Murcia, Spain; cdquero@ucam.edu; 3 Faculty of Sport, UCAM Catholic University of Murcia, 30007 Murcia, Spain; 4 Faculty of Education, University of Zaragoza, 50009 Zaragoza, Spain; dlplazapal@unizar.es

**Keywords:** respiratory muscles, scuba diving, performance, fatigue, dyspnea, gas tank

## Abstract

**Background/Objectives**: The inspiratory muscles play a fundamental role in cardiorespiratory performance, especially in water sports. The main objective of this study was to investigate the effects of an inspiratory muscle warm-up (IMW) protocol on the performance and respiratory parameters of scuba divers prior to performing two diving tests, one static and the other dynamic. **Methods**: Eight young, active divers (six men and two women; 26.63 ± 4.67 years of age) volunteered for the study. In two sessions, one using an IMW protocol and the other without IMW, participants performed two underwater tests with a gas tank: a static immersion at the bottom of the pool for 5 min and a dynamic test consisting of swimming underwater for 12 min at 1 m/s. Some cardiorespiratory parameters such as gas expenditure, oxygen saturation (SO_2_), heart rate (HR), spirometry, and rating of perceived exertion (RPE) were measured before and after each of the tests. **Results**: Significant differences were observed in gas expenditure using IMW in both static and dynamic testing (*p* < 0.05) and in RPE in dynamic testing (*p* < 0.05). HR values were significantly higher in the pre-dynamic test with IMW and the post-static test with IMW. Finally, with the use of IMW, spirometry values were significantly higher (*p* < 0.05) in static tests after immersion, whereas in the dynamic test, they were significantly higher before the immersion (*p* < 0.05). **Conclusions**: Based on the results of this study, the use of the IMW prior to a dive would be recommended for better overall physical performance, oxygen expenditure, improved pulmonary function, and lower perceived exertion.

## 1. Introduction

Diving is an underwater activity undertaken for recreational, sporting, or research purposes, where individuals submerge using breathing or non-breathing systems [1]. According to Pendergast and Lundgren [2], diving poses challenges related to adapting to the aquatic environment, primarily due to the thorax’s limited capacity and the need to manage hyperoxia and hypercarbia. Effective breathing control is crucial in diving, as it optimizes gas consumption, buoyancy, and relaxation. As hydrostatic pressure increases, both air consumption and respiratory effort rise, leading to respiratory fatigue [2]. Trained divers, however, experience less fatigue and better oxygen conservation compared to untrained divers [3].

The monitorization of cardiorespiratory variables is paramount in the pursuit of enhancing diving performance [2,4,5]. Martín-Escudero et al. [6] emphasized that oxygen saturation is a valuable tool for assessing sport performance during exercise. Peng et al. [7] studied apnea in divers, underscoring the importance of forced spirometry in optimizing performance. Additionally, heart rate (HR) is regarded as a key indicator of exertion during diving, as it enables divers to monitor their pulse, increase safety, and manage their workload more effectively [8].

The systematic training of inspiratory muscles has a positive impact not only on patients with cardiorespiratory issues [9] but also on overall sports performance [10,11,12]. In recent years, there has been a growing interest in the use of IMW in sports. This technique has been shown to improve recovery after high-intensity exercise [5]. Initially applied in patients with chronic diseases [13], IMW has since been adopted by endurance athletes and team sports players, contributing to improved cardiorespiratory and muscular performance [14,15,16]. Tanriverdi et al. [17] explored the effects of IMW on the autonomic nervous system and arterial stiffness, concluding that this warm-up optimizes physiological parameters in athletes. In endurance sports, IMW has also been linked to increased tissue oxygenation, which enhances physical performance [18].

Recent studies have further indicated that performing an IMW prior to physical activity boosts the neuromuscular activity of inspiratory muscles [14,19]. Cirino et al. [14] reviewed 31 studies on IMW as a warm-up strategy, highlighting its performance-enhancing effects in aquatic sports, such as swimming [20]. However, its effects on cardiorespiratory variables during diving remain unexamined, and evidence is still limited across some sports.

Therefore, this pioneering study aimed to evaluate whether performing an IMW before diving with scuba equipment can reduce gas expenditure and respiratory effort, thereby improving oxygen conservation. Specifically, it will investigate the acute effects of IMW on diving performance by analyzing its impact on gas consumption, pulmonary function, perceived exertion, and oxygen saturation.

## 2. Materials and Methods

### 2.1. Participants

A total of 8 participants (6 males and 2 females) took part in this descriptive observational study on a voluntary basis (age: 26.63 ± 4.67 years; weight: 72.4 ± 17.54 kg). The inclusion criteria were (a) performing at least 150–300 min per week of physical activity at a moderate to vigorous intensity; (b) had previously performed at least 3 immersions using a gas tank; and (c) having a minimum of one year of scuba diving experience (mean experience: 2.91 ± 1.78 years). As exclusion criteria, participants under 18 years of age and those with any medical contraindication for diving were rejected.

Participants were informed of the study objectives and procedures following the Declaration of Helsinki ethical principles for medical research involving human participants, and all signed written informed consent prior to the start of the experiment. The University Ethics Committee approved the study protocols and procedures prior to the study (UALBIO2024/026).

### 2.2. Procedures

**Figure 1:**
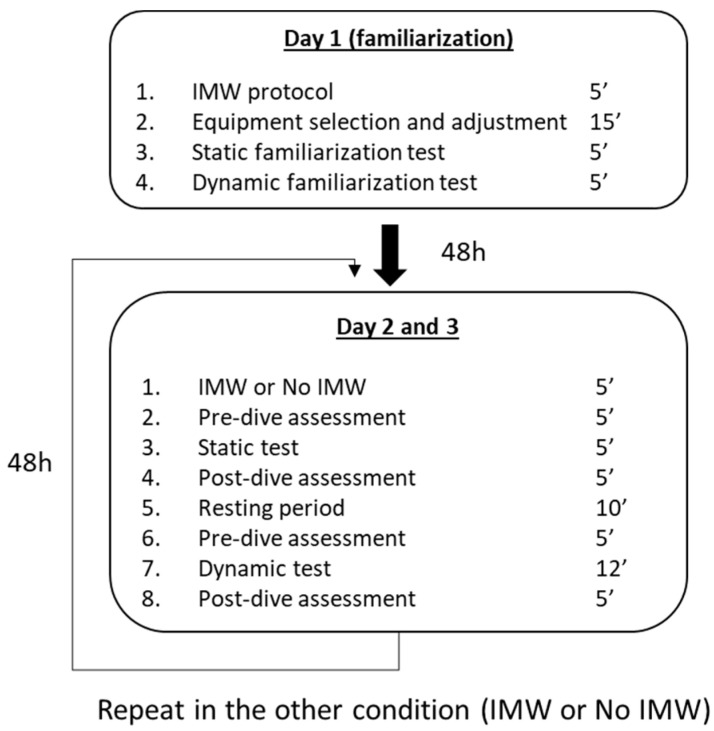
Schematic diagram of the study protocol.

All measurements were taken in a 25 m long pool at a depth of between 1.50 and 2.10 m and at an average temperature of 28.5 °C. The study took place during 3 non-consecutive sessions at least two days apart. The study protocol is summarized in Figure 1. The first session consisted of a familiarization with performing an IMW protocol. Subsequently, they proceeded to a static and a dynamic dive of 5 min each with the diving equipment (fins, jacket, mask, regulator, and gas tank of approximately 200 Bars) they were comfortable with. Participants were asked to use the same equipment for all immersions along with testing. In addition, for the static dive, they wore a weight belt to facilitate the immersion. Participants were urged not to exercise strenuously or consume caffeine, alcohol, or tobacco at least 12 h before the start of the study.

The IMW condition was randomly assigned to each participant for the second or third session. In these sessions, participants started with the IMW, if applicable, and then pre-dive values (pre) of gas expenditure, spirometry, oxygen saturation, heart rate, and RPE were measured. Subsequently, the static immersion test was performed, and just after finishing, the same parameters were measured (post). Finally, after a 10 min rest to ensure recovery, the dynamic immersion test was performed, and the same parameters were measured pre- and post-immersion.

#### 2.2.1. Inspiratory Muscle Warm-Up (IMW)

The IMW was performed using the Powerbreathe^®^ Classic Competition (Biolaster, Andoain, Spain). Following the recommendations of Nepomuceno Junior et al. [18], participants performed under the supervision of a researcher 30 breaths under inspiratory resistance, equivalent to 56% of maximal inspiratory pressure (MIP). Participants were asked to perform the breaths just prior to performing the static test on the assigned day.

#### 2.2.2. Immersion Protocols: Static and Dynamic Immersion Test

**Figure 2:**
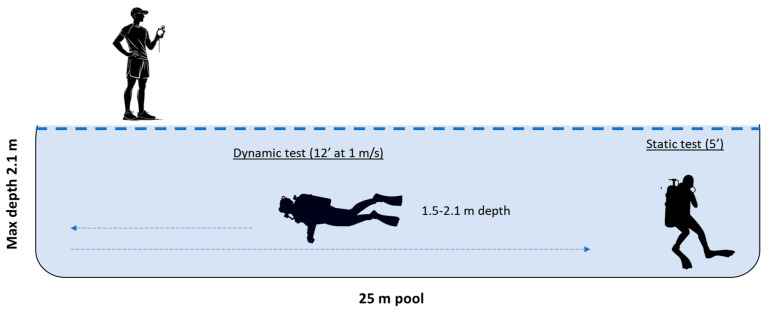
Diagram of the study procedures of the static and dynamic tests.

The tests procedures are detailed in Figure 2. First, a static immersion test was performed following the procedures of Peng et al., [7] in which participants had to maintain deep and controlled breathing for 5 min at the bottom of the pool; they could move, but in no case perform any kind of swimming. The dynamic immersion test consisted of a 12 min swim at 1 m/s. By means of acoustic signals, an instructor indicated the swimming pace to the participants as they passed every 25 m.

### 2.3. Outcomes

#### 2.3.1. Gas Consumption

The calculation of gas consumption during a dive was carried out using a manometer, an instrument that indicates the number of bars available inside the gas tank. To calculate the gas consumption during our study, we used a TEKNON pressure gauge with a 400 BAR flexible tester (Tecnomar, Valencia, Spain). To obtain gas consumption during the dive, the pre and post values were noted down to obtain the difference between the two.

#### 2.3.2. Forced Spirometry (FS)

This test was performed using a Vitalograph spirometer (Mecanmed, Guangzhou, China) to obtain the values of forced spirometry. Participants were instructed to exhale a maximum of three times. The best of three attempts, allowing at least 1 min rest in between, was considered for subsequent analysis.

#### 2.3.3. Oxygen Saturation (SO_2_) and Heart Rate (HR)

Oxygen saturation and heart rate were measured pre- and post-immersion using a BRAUN 1 pulse oximeter (Braun, Kronberg, Germany). The device was placed on the index finger of the participant’s right hand. The post-measurement was conducted following a 3 min rest period for the participants.

#### 2.3.4. Rate of Perceived Exertion (RPE)

The Borg scale or RPE was used after the proposed dives (static and dynamic). Participants were asked to rate their effort using an adapted scale of 0 to 10 (OMNI scale) [21].

### 2.4. Statistical Analysis

All statistical analyses were performed using the Statistical Package for the Social Sciences v24.0 (SPSS Inc., Chicago, IL, USA). Measures of homogeneity and dispersion were described as mean ± standard deviation (SD). The Shapiro–Wilk test was used to test the hypotheses of normality and homogeneity of variance. The effects of IMW were examined through a two-way ANOVA of repeated measures using the general linear model with 2-time points × 2 groups (No IMW and IMW). To allocate the differences when significant effects in ANOVA were determined, post-hoc test (Bonferroni) was performed. Partial eta squared (η^2^p) for variance analysis was used for the identification of the effect size, considering small effect values between 0.1–0.24, medium effect between 0.25–0.36, and large effect ≥0.37 [22]. The level of statistical significance was set at *p* < 0.05. The effect size of the observed differences was analyzed using Cohen’s d and was considered small when values ranged between 0.2 and 0.5, moderate when values ranged between 0.5 and 0.8, and large when the effect was >0.08 [22].

## 3. Results

Table 1 summarizes the differences in the cardiorespiratory parameters analyzed during the static immersion test with and without the use of IMW. When using IMW, significantly greater values (*p* < 0.05) were observed for gas consumption, HR, and FS in Post-tests in comparison to No IMW. Regarding effect size, Cohen’s d was considered large (*d* > 0.8) for both gas expenditure and HR measurements. In addition, time effect analysis revealed significant differences in O_2_ saturation between pre- and post-dive tests for the IMW condition (*p* < 0.05).

Table 2 presents the differences in cardiorespiratory parameters analyzed during the dynamic immersion test with and without the use of IMW. Significantly greater values (*p* < 0.05) were observed for gas expenditure, pre-dive HR, pre-dive FS, and RPE when IMW was performed. Moderate effect sizes were found for FS and gas expenditure, while large effect sizes were observed for HR and RPE. In both conditions, significant time effects were determined in O_2_ saturation between pre- and post-dive tests (*p* < 0.001). However, the HR examination only revealed significant differences when no IMW was applied.

## 4. Discussion

The purpose of this study was to investigate the acute effects of performing an inspiratory muscle warm-up on scuba divers’ overall physical performance and key cardiorespiratory variables during static and dynamic immersion tests.

The main finding of this study indicates that IMW improves overall physical performance and cardiorespiratory parameters, with a significant reduction in gas expenditure during both static and dynamic tests when using the IMW. This effect could primarily be due to improved airway efficiency and pulmonary function from using the IMW via Powerbreathe^®^.

The IMW enhances both general and respiratory performance during exercises with high oxygen demands, such as scuba diving. Additionally, a significant increase in pulmonary function (spirometry) was found in the IMW pre-test.

In a previous study, Kilding et al. [23] implemented a 6-week respiratory muscle training protocol and observed greater maximal inspiratory pressure (MIP) and improved performance in 100 m and 200 m swimming, supporting its inclusion in training programs for high-performance swimmers. While MIP is a valuable metric, IMW alone already enhances it, so in this study, it was deemed more relevant to focus on pulmonary function (maximal expiratory pressure) measured through spirometry, as this is a key performance indicator in underwater activities [5].

Most investigations into IMW in sports have shown significant increases in inspiratory function without significant changes in spirometry [14]. However, no studies have examined the effects in aquatic environments, where breathing dynamics differ due to pressures on the rib cage. The expansion of the rib cage with IMW may be linked to the improvements in pulmonary function observed in this research, as well as enhanced oxygen exchange in the airways [10]. In their study, Carvajal-Tello et al. [24] indicated that during vigorous exercise (dynamic test), the demands on the respiratory system increase significantly, as it acts as a metabolic buffer that increases the strength of the respiratory muscles to ensure ventilation. If these muscles are inefficient, the muscles will become more fatigued because the removal of metabolites cannot keep up with the oxygen production rhythm. In addition, Koizumi and Ohya [25] determined that IMW in prolonged dynamic efforts improves respiratory function and muscle oxygenation due to greater ventilatory and airway efficiency of the inspiratory muscles. Conversely, if these muscles, mainly the diaphragm, have a greater capacity for contractile response and force generation, it could result in a better tolerance to fatigue and a lower perception of dyspnea during underwater exercise [26]. Consequently, this would trigger a substantially lower gas expenditure during IMW dives.

Regarding training adaptations in spirometric values, Peng et al. [7] found that apnea-trained divers respond better to diving and that forced spirometry contributes more significantly to performance than other variables. In the current investigation, forced spirometry values tended to increase with IMW, especially during static diving. This may be because fatigue had a greater impact on dynamic test parameters.

The decision to conduct the static test in the first place was due to the absence of accumulated fatigue that otherwise would have influenced subsequent tests. The application of the static test for 5 min was based on the procedures described by Peng et al. [7], who found significant spirometry and cardiorespiratory changes when implementing this static apnea during that time. On the other hand, the dynamic test was performed for 12 min as an adaptation of the Cooper Test described by Ojeda et al. [26], which represents a good indicator of cardiopulmonary function at constant speed.

In a related study, Costalat et al. [3] concluded that after a 6-week dive training program, trained divers showed greater efficiency in oxygen conservation, sensitivity to oxygen, and forced expiratory capacity during dynamic dives. These findings underscore the importance of understanding gas expenditure with IMW, regardless of diving skill level, as well as forced spirometry values. In the present research, both gas expenditure and the pre-dynamic FS test revealed significant improvements with IMW. The post-static FS test also yielded significant results.

Slight reductions in SO_2_ (about 4%) have been observed during diving, indicating significant biological oxygen conservation, typically maintained during constant inhalation [4]. However, no significant results were found here, though a tendency toward better oxygen conservation with IMW during prolonged efforts (dynamic test) was observed. Further studies are needed to confirm this trend, which may be due to improved oxygen transport with IMW, as discussed above.

In their study, De Asís-Fernández et al. [5] analyzed recovery after high-intensity interval training (HIIT), observing that IMW with 30 breaths at 56% of MIP improved recovery time by 3 s compared to no IMW. The findings identified in the current study revealed an increase in heart rate (HR) when using IMW in both static and dynamic immersion tests. This could be attributed to fatigue, exertion, or increased inspiratory muscle engagement due to IMW. However, perceived exertion (RPE) was lower with IMW, suggesting that IMW may have reduced the effort and improved tolerance by reducing respiratory muscle resistance through rib cage expansion.

Cirino et al. [14] conducted a systematic review on the effects of IMW on both cardiorespiratory and performance parameters. Positive effects were reported in 88% of studies for respiratory parameters and 44% for performance parameters, likely due to variations in protocols and intensities across studies. Standardized studies with consistent repetitions, intensities, and respiratory loads are needed to confirm the positive effects of IMW. Nonetheless, the traditional protocol of 30 breaths at around 40% of MIP improves physical performance during high-intensity exercise, enhances respiratory muscle contraction efficiency, reduces dyspnea, and improves performance by leveraging the contractile and biochemical properties of inspiratory muscles, motor units, and muscle fibers.

Most studies focus on IMW as a tool to improve the quality of life for people with chronic diseases. However, previous research revealed that IMW enhances inspiratory muscle neuro-muscular function, increases PIM, and improves athletes’ perceived exertion [16,19,27]. This aligns with our findings, where RPE values were lower with IMW in both static and dynamic immersion tests, suggesting a reduction in effort.

One of the key questions of the study was the acute effect of IMW on HR at different intensities. Tanriverdi et al. [17] reported a decrease in HR at 10% IMW, alongside positive changes in blood pressure and autonomic function, while arterial stiffness increased at 60% IMW, resulting in favorable changes in physical and cardiorespiratory performance.

IMW is presented as an innovative and effective tool for improving airway efficiency and overall physical performance [11]. Based on these findings, it can be suggested that IMW performed prior to a scuba diving immersion may have acute, positive effects on overall physical performance and cardiorespiratory variables in divers. However, given the small sample size, further research is needed to confirm these results. IMW could also help physical activity and sports professionals design training programs that positively impact lung function, maximal inspiratory strength, muscle oxygenation, and performance in healthy athletes. According to Lorca Santiago et al. [28], the ideal protocol would involve two consecutive sessions of 30 breaths at 40% of MIP, with a one-minute rest between sessions, to reduce fatigue, dyspnea, and the metaboreflex.

The practical application of IMW could be highly beneficial for novice divers who struggle with correct regulator use or professional divers who need to manage gas consumption efficiently during extended dives. Additionally, the reduction in effort and enhanced sense of calm may benefit divers prone to stress.

The main limitations of this study were the small sample size, the inability to conduct the study in open water, the low number of women scuba divers, and restricted diving equipment that allowed only paired testing. In addition, the water temperature in this study was typically higher than in natural conditions. Thus, the comparison with other studies should take this factor into consideration since the cardiorespiratory demands might be different. Future research should investigate higher intensities (around 80–85% of MIP) and examine the effects on various respiratory parameters, as well as IMW’s application in longer-duration dives and the identification of potential gender differences. It would also be important to measure maximum voluntary ventilation, a key predictor of oxygen conservation, which could not be measured in this study due to the cost of equipment.

## 5. Conclusions

This study is the first to analyze the acute effects of an IMW protocol on performance and cardiorespiratory variables prior to scuba diving immersion using a gas tank. IMW has proven to be an effective strategy for enhancing both overall physical performance and cardiorespiratory parameters in divers. Lower gas expenditure was observed with the IMW during both static immersion and dynamic tests at a moderate effort. Forced spirometry values, however, were consistently higher with IMW in both static and dynamic tests across all participants. Significant improvements were observed in post-test spirometry during the static test and pre-test spirometry during the dynamic test with IMW. Based on these findings, it can be suggested that IMW performed prior to a scuba diving immersion may have acute, positive effects on overall physical performance and cardiorespiratory variables in divers. However, given the small sample size, further research is needed to confirm these results.

## Figures and Tables

**Table 1:** Physiological parameters for static immersion according to the inspiratory muscle warm-up.

		NO IMW	IMW			ANOVA								
						Time Effect			IMW Effect			Time × IMW Effect		
		Mean ± SD	Mean ± SD	*p*	*d*	F	*p*	η^2^p	F	*p*	η^2^p	F	*p*	η^2^p
Gas consumption (bar)		13.75 ± 3.77	10.00 ± 2.31	0.034	1.19									
O_2_ saturation (%)	Pre	98.12 ± 0.99	98.37 ± 0.74	0.458	0.28	7.364	0.030	0.513	1.647	0.240	0.190	0.563	0.478	0.074
	Post	95.00 ± 4.27	96.62 ± 1.50 *	0.233	0.5									
Spirometry (L/min)	Pre	567.50 ± 185.40	620.00 ± 150.02	0.73	0.31	0.312	0.594	0.043	18.152	0.004	0.722	6.081	0.063	0.326
	Post	539.37 ± 203.72	600.00 ± 161.75	0.025	0.33									
HR (bpm)	Pre	84.25 ± 12.57	90.00 ± 9.70	0.293	0.51	0.246	0.635	0.034	10.112	0.013	0.714	5.341	0.054	0.433
	Post	78.12 ± 7.39	94.00 ± 11.04	0.005	1.69									
RPE		2.37 ± 2.72	1.50 ± 1.60	0.059	0.3									

IMW: respiratory muscle warm-up; HR: heart rate; BPM: beats per minute; RPE: rate of perceived exertion. * Significant differences with respect to pre-dive (*p* < 0.05).

**Table 2:** Physiological parameters for dynamic immersion according to the inspiratory muscle warm-up.

		NO IMW	IMW			ANOVA								
						Time Effect			IMW Effect			Time × IMW Effect		
		Mean ± SD	Mean ± SD	*p*	*d*	F	*p*	η^2^p	F	*p*	η^2^p	F	*p*	η^2^p
Gas consumption (bar)		59.37 ± 15.90	51.25 ± 15.05	0.001	0.526										
O_2_ saturation (%)	Pre	96.37 ± 3.15	96.12 ± 2.79	0.888	0.008	177.144	<0.001	0.962	0.012	0.915	0.002	0.732	0.420	0.095	
	Post	92.25 ± 3.10 *	93.12 ± 3.97 *	0.345	0.244										
Spirometry (l/min)	Pre	561.25 ±176.97	625.00 ± 142.60	0.006	0.56	1.714	0.232	0.197	37.741	0.003	0.872	6.581	0.052	0.349	
	Post	563.75 ± 155.35	590.00 ± 167.90	0.121	0.44										
HR (bpm)	Pre	81.25 ± 10.01	91.37 ± 10.01	0.001	1.01	10.915	0.013	0.609	16.024	0.005	0.696	0.048	0.834	0.007	
	Post	87.37 ± 14.16 *	96.62 ± 8.99	0.054	0.77										
RPE		4.37 ± 1.30	5.56 ± 1.29	0.012	0.91										

IMW: Respiratory muscle warm-up; HR: heart rate; BPM: beats per minute; RPE: rate of perceived exertion. * Significant differences with respect to pre-dive (*p* < 0.05).

## Data Availability

The datasets are available upon reasonable request from the corresponding authors.

## Abbreviations

The following abbreviations are used in this manuscript:

IMW	inspiratory muscle warm-up
SO_2_	oxygen saturation
RPE	rate of perceived exertion
HR	heart rate
MIP	maximal inspiratory pressure
FS	forced spirometry
BPM	beats per minute
